# Knomics-Biota - a system for exploratory analysis of human gut microbiota data

**DOI:** 10.1186/s13040-018-0187-3

**Published:** 2018-11-06

**Authors:** Daria Efimova, Alexander Tyakht, Anna Popenko, Anatoly Vasilyev, Ilya Altukhov, Nikita Dovidchenko, Vera Odintsova, Natalya Klimenko, Robert Loshkarev, Maria Pashkova, Anna Elizarova, Viktoriya Voroshilova, Sergei Slavskii, Yury Pekov, Ekaterina Filippova, Tatiana Shashkova, Evgenii Levin, Dmitry Alexeev

**Affiliations:** 1 Research and Development Department, Knomics LLC, Skolkovo Innovation Center, Moscow, Russian Federation; 2 Computer Technologies Laboratory, ITMO University, Saint Petersburg, Russian Federation; 3 Faculty of Biological and Medical Physics, Moscow Institute of Physics and Technology (State University), Moscow, Russian Federation; 4 Life Sciences Department, Skolkovo Institute of Science and Technology, Moscow, Russian Federation; 5 Biology Department, Lomonosov Moscow State University, Moscow, Russian Federation; 6 Institute of Cytology and Genetics, Novosibirsk State University, Novosibirsk, Russian Federation; 7 Institute of Protein Research, Russian Academy of Sciences, Pushchino Moscow, 142290 Russia

**Keywords:** Metagenome, Microbiome, Web service, Bioinformatic pipeline

## Abstract

**Background:**

Metagenomic surveys of human microbiota are becoming increasingly widespread in academic research as well as in food and pharmaceutical industries and clinical context. Intuitive tools for investigating experimental data are of high interest to researchers.

**Results:**

Knomics-Biota is a web-based resource for exploratory analysis of human gut metagenomes. Users can generate and share analytical reports corresponding to common experimental schemes (like case-control study or paired comparison). Interactive visualizations and statistical analysis are provided in association with the external factors and in the context of thousands of publicly available datasets arranged into thematic collections. The web-service is available at https://biota.knomics.ru.

**Conclusions:**

Knomics-Biota web service is a comprehensive tool for interactive metagenomic data analysis.

**Electronic supplementary material:**

The online version of this article (10.1186/s13040-018-0187-3) contains supplementary material, which is available to authorized users.

## Background

The last decade was marked by an explosive growth of experimental data characterizing human-associated microbial communities using metagenomic approach. Previously utilized mainly by the academic community, now metagenomics are used in the industry to assess structure, functions and dynamics of microbiota composition - particularly, to identify the impact of change in diet and medications intake on human microbiota and health. Visual and statistical exploration of important functions of microbiota (like antibiotic resistance [1] and dietary fiber catabolism [2, 3]) is of particular importance in the global context of publicly collected data. Lower costs and increasing popularity make metagenomics further available to smaller companies and research facilities that often lack dedicated staff bioinformaticians that can perform manual statistical analysis and insight-providing visualization according to state-of-art guidelines [4, 5]. In order to optimize the translation of metagenomic surveys’ results into biomedically important knowledge and advance the global progress in collaborative microbiota research, we developed Knomics-Biota, a web-service for metagenomic data analysis that allows users without advanced skills in bioinformatics and software development to turn their “raw” data into intuitive analytical reports. The datasets can be accompanied with metadata that can include, besides factors like age and clinical status, the factors related to experimental design - distribution between case and control groups, paired correspondence of the samples, etc. After automatic analysis is complete in the cloud, a user is provided with online reports describing all steps of metagenomic analysis - from data quality check and composition profiles to statistical hypothesis testing. Interactive visualization modules allow to explore the interactions between microbiota and factors in detail and propose novel biological hypotheses. Analysis of metabolic potential includes manually curated pathways reflecting gut microbiota functions highly relevant for human health - like synthesis of short-chain fatty acids (SCFAs) and vitamins. It is possible to analyze one’s own data in the context of related precomputed published metagenomes arranged into collections (diet, inflammatory bowel diseases, world populations, etc.). The generated reports can be shared privately with collaborators or publicly and readily to be referred to in scientific publications.

## Implementation

The computational backend of the system is located in the cloud (Additional file 1: Figure S1) and makes use of publicly available software solutions. The front-end interface of the web service is implemented using Yii framework, and interactive visualisations are based on d3js library. The web-service is available at the address: https://biota.knomics.ru. After signing up, a user can upload one’s own metagenomic read sets (obtained using 16S rRNA or “shotgun”/WGS [whole genome] sequencing) accompanied with data description files (metadata).

**Fig. 1:**
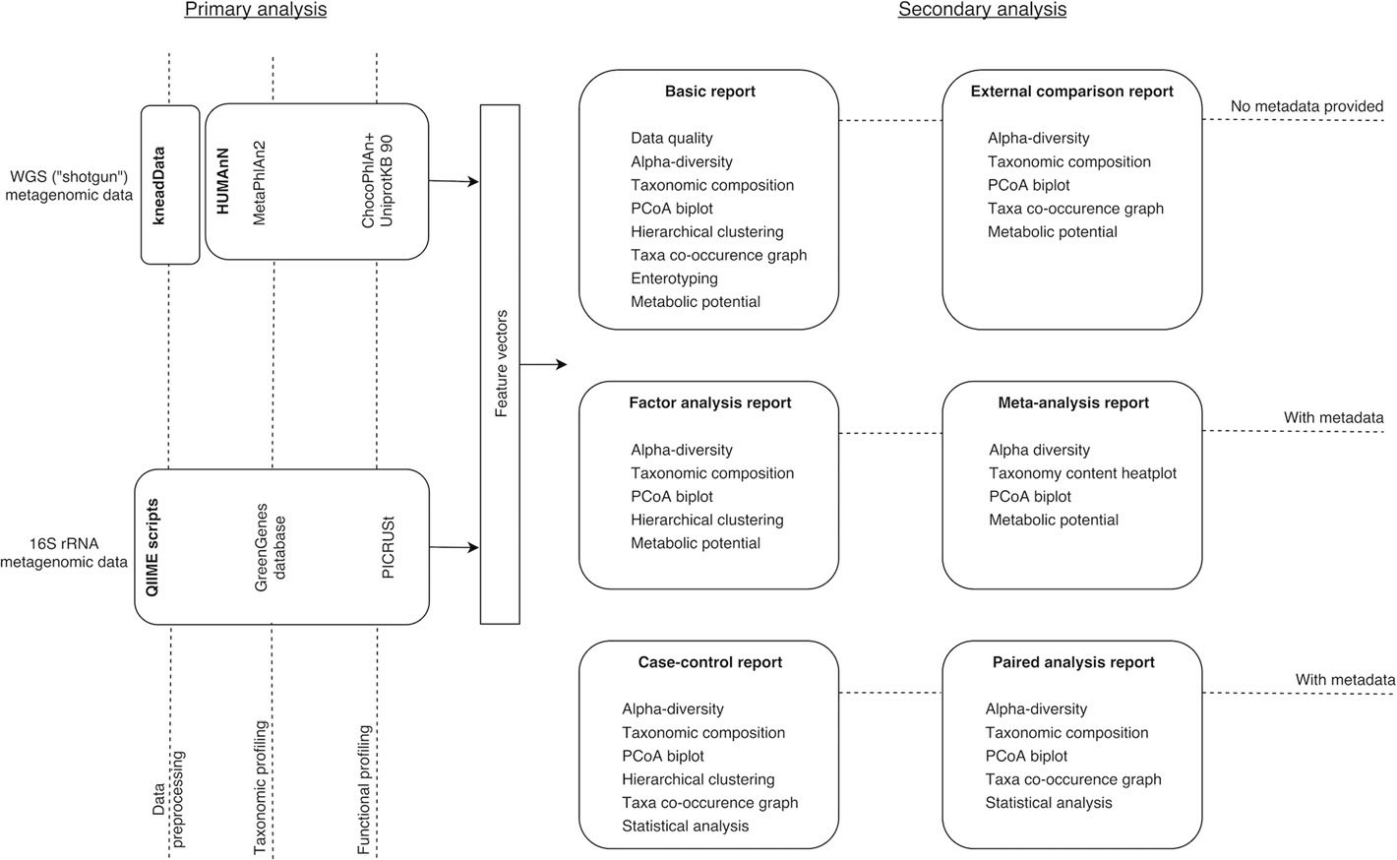
Workflow of the Knomics-Biota web-service. The workflow is split into two basic steps: primary and secondary analysis, for both amplicon and WGS metagenomic data

General logic of Knomics-Biota service includes two components: primary and secondary analysis (Fig. 1). The primary analysis component encompasses basic processing of the reads to obtain microbiota composition profiles. For each of the 16S rRNA and WGS formats, primary analysis component produces feature vectors including relative abundance of microbial taxa at various ranks as well as of gene groups and metabolic pathways according to KEGG Orthology and Enzyme Commission (EC) nomenclatures. Additionally, some functions are analyzed in a dedicated way due to their importance for human health - synthesis of vitamins and SCFAs. These functions are assessed for each sample using curated pathways (Additional file 2: Figure S2).

The primary analysis of 16S rRNA data is performed using QIIME [6], from reads filtering to defining OTUs (operational taxonomic units). Gene content is predicted using PICRUSt algorithm [7]. WGS data is analyzed using KneadData for quality filtering and HUMAnN [8] - for taxonomic and functional profiling.

The secondary analysis component implemented in Python v. 3.2 includes statistical analysis of the feature vectors (together with the metadata, if provided) and generating static figures as well as input (in JSON format) for interactive visualization modules. The workflow of the secondary analysis varies depending on the choice of report type by the user (see Fig. 1).

The Basic report is generated initially for any user data. It includes quality check of the “raw” data, assessment of relative abundance of taxa and functional gene groups as well as alpha-diversity. Hierarchical clustering, enterotyping [9] and metabolic potential prediction are performed. Besides the basic visualizations, interactive modules are provided including heatmap, PCoA (principal coordinates analysis) plot, alpha-diversity plot and co-occurrence network [10]. Each module within Basic and other interactive reports of Knomics-Biota is accompanied with the details of implementation (algorithm and databases used, values of control parameters, etc) so that a user is able to replicate the results independently - as well as to describe the methods in one’s scientific publication.

The bioinformatic algorithms in the secondary analysis include PERMANOVA method for multivariate analysis, regression linear models and U-test for discovering links between microbial features and factors. Outliers are identified using Grubbs’ test and removed from further statistical analysis. Multiple testing adjustment is performed using Benjamini–Hochberg procedure.

## Results and discussion

A number of metagenomic analysis pipelines have been developed. They vary in analysis options - by providing only primary “raw” data processing or advanced options as well, allowing different input data formats (16S rRNA sequencing or WGS data). A comparison data is provided in Table 1 highlighting that Knomics-Biota provides a rich repertoire of functions making it superior to alternatives. As seen, only Knomics-Biota and MG-RAST [11] provide databases of published metagenomes for comparative analysis. Nephele [12] as well as CosmosID and One Codex platforms provide a similar functionality: “raw” data processing, advanced statistical analysis and visualizations. However, none of them provide interactivity enabling to change parameters of display on-the-fly.

**Table 1:** Comparison of Knomics-Biota functionality with other pipelines

Pipeline name	“Raw” data analysis		Statistical analysis			External datasets availability	Data sharing
	16S rRNA sequencing	WGS	Basic statistics	Group comparison	Interactive Visualizations		
Knomics-Biota	Yes	Yes	Yes	Yes	Yes	Yes	Yes
Nephele	Yes	Yes	Yes	Yes	No	Yes (data from HMP [16] only)	Yes
MG-RAST	Yes	Yes	Yes	No	No	Yes	Yes
One Codex	No	Yes	Yes	Yes	No	No	Yes
GUSTA ME	No	No	Yes	Yes	No	No	No
CosmosID	No	Yes	Yes	Yes	No	No	Yes
QIAGEN Microbial Genomics Pro Suite	No	Yes	Yes	No	No	No	NA
Calypso	Yes	No	Yes	Yes	No	No	No

The Knomics-Biota is made free for academic use. For commercial use, special licensing is provided. Time of the free analysis depends on the number of projects in the queue and is likely to change during the evolution of the system, but currently, an analysis of a typical 16S rRNA dataset containing around 100 samples from a single Illumina MiSeq run (as a prevalent input data format) is processed within several hours. Overall, as much as approximately 5000 of 16S rRNA samples can be submitted at once by a user. As for the WGS analysis, due to the high data volume and queue the processing can take longer - for example, around several days for 50–100 WGS metagenomes.

Before starting to upload one’s own data to Knomics-Biota, it is possible to get a glance into the complete set of functions on existing datasets. After logging in anonymously into a demo account, a user is provided with sample analytical reports precomputed for publicly available metagenomic data with meta-data from several large-scale studies examining microbiome in various conditions like colon cancer [13], inflammatory bowel diseases [14] and malnutrition [15] as well as associated with dietary interventions [3]. The list of the external datasets is being regularly updated with newly published metagenomes related to human gut microbiota (as well as other niches).

After signing up and logging in, a user can create a project in his/her account and upload the “raw” data - metagenomic reads in FASTQ format obtained via amplicon (16S rRNA) or WGS. When the uploading process is finished, a user can go on with the analysis - always starting with the Basic report. Unlike the other reports, the Basic report generation does not require neither the metadata nor specification of external context. The report includes the results of quality check, microbiota taxonomic and functional composition profiling and alpha-diversity. Similar existing services often require complex configuration steps from a user, provide only basic analysis functionality [6] or are highly specialized [1]. After the Basic report has been successfully generated, it is possible to perform advanced analysis. The major report types and their contents are briefly shown in the Fig. 1.

One of the essential functions of Knomics-Biota is the opportunity to analyze user data in the context of thousands of metagenomes from publicly available articles precomputed using the same pipeline. The collection of external datasets is regularly updated. For convenience, they are arranged into collections (contexts) according to their topic. The major microbiota topics include inflammatory bowel diseases (IBD), diet, fecal mass transplantation (FMT), antibiotics, world populations, Parkinson’s disease, and so on. Accordingly, while it is possible to compare one’s own data against all metagenomes in Knomics-Biota database, it is often reasonable to limit the analysis to the relevant context - using the External comparison report (without user metadata) or Meta-analysis report (with user metadata provided). When the analysis is complete, a user is notified via email.

When the information on the membership of each samples in case or control group is uploaded, the corresponding Case-control report becomes available - allowing to compare these datasets statistically and visually - similar to the scenario of External comparison. The functionality of interactive modules is extended to allow comparison of the microbiota composition between the two groups. Statistical analysis is performed to identify the respective significant differences. Besides the basic composition features, gut microbiota-specific characteristics of interest are evaluated and compared between the groups: these include metabolic potential for synthesis of vitamins and SCFAs. Paired analysis report has a workflow similar to a case-control scenario but modified to account for paired type of data (for instance, the metagenomes obtained from the same subjects before and after antibiotic therapy).

A Factor analysis report is generated if metadata with extrinsic/intrinsic factors is provided. The service performs multifactor analysis to identify significant associations between microbiota composition and factors like age, body-mass index (BMI), clinical status, etc. The interactive modules are extended to include controls over the display of these factors aiding in exploratory analysis. Additionally, a separate type - Time series report - is dedicated to the examination of consecutively grouped samples including specific algorithms like taxon stability analysis and visualizations of these points.

To facilitate collaborative research, Knomics-Biota allows to adjust access control. By default, the uploaded data and generated reports are only visible to the user. However, it is possible to share any of the reports globally in view-only mode (using a permanent link) or to share the project privately to collaborators registered in the service.

## Conclusions

Knomics-Biota service is a convenient tool for collaborative exploratory analysis of metagenomes in the context of publicly available data. Thematic collections of metagenomes focused on microbiota in specific diseases and of world populations, the impact of dietary and medical interventions are useful for comparative surveys and data validation. Besides gut microbiota, the system is ready for processing metagenomes from an arbitrary environment allowing users with and without expertise in bioinformatics to gain insights into system biology of complex microbial communities.

## Availability and requirements

Project name: Knomics-Biota.

Project home page: https://biota.knomics.ru

Operating system(s): Platform independent.

Programming language: Python.

Other requirements: browser, Internet connection.

License: GNU GPL.

Any restrictions to use by non-academics: academic use is free; for commercial use, licensinge is required.

## Additional files

Additional file 1: **Figure S1.** An architecture of Knomics-Biota web service. (PDF 31 kb)

Additional file 2: **Figure S2.** Manually curated vitamin biosynthesis pathways used in the analysis. (PDF 1598 kb)

## Abbreviations

SCFA: Short Chain Fatty Acid
WGS: Whole Genome Sequencing
