# Supplementary figures and images for: Knomics-Biota - a system for exploratory analysis of human gut microbiota data
Source: BioData Min. 2018 Nov 6;11:25. doi: 10.1186/s13040-018-0187-3 (PMC6220475; doi:10.1186/s13040-018-0187-3)

# Knomics / biota infrastructure

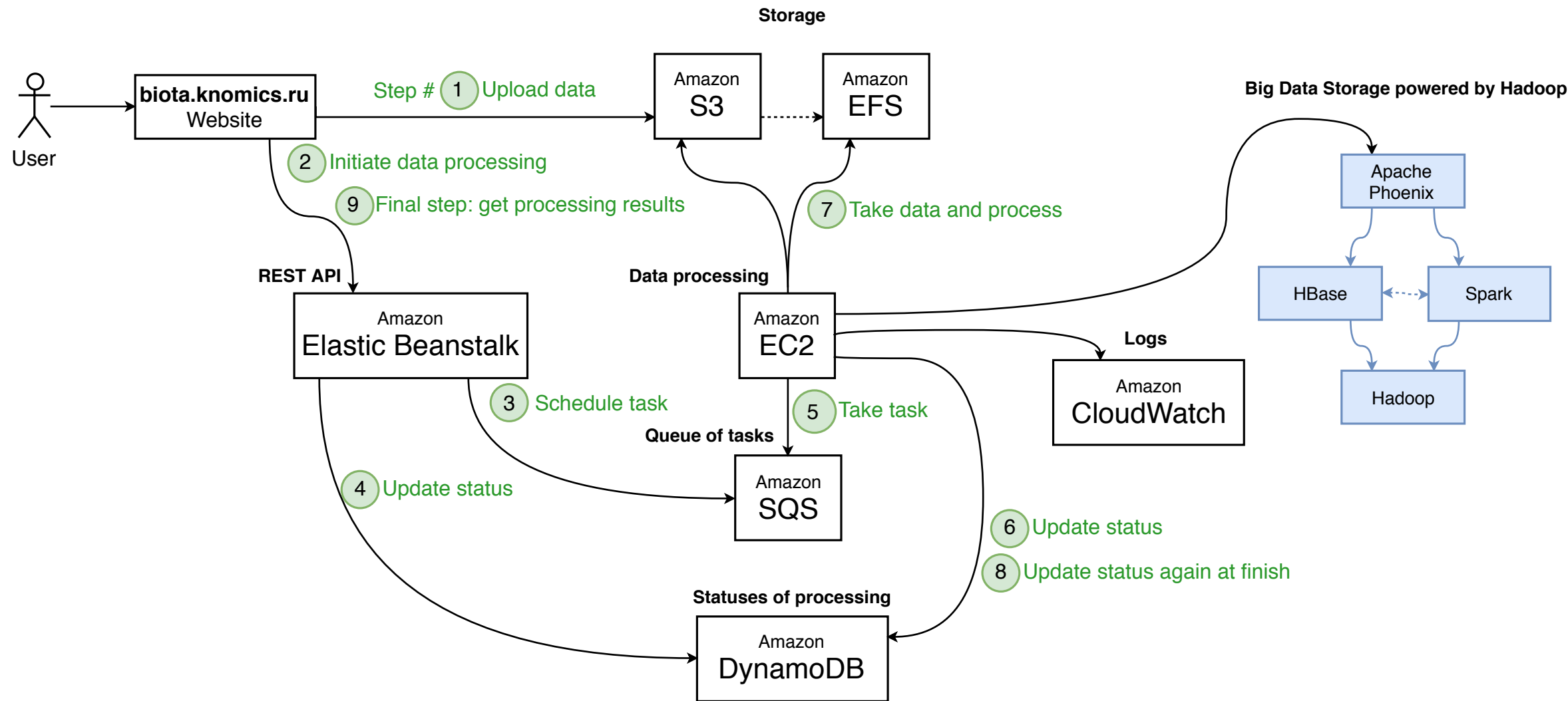

Supplement: Supplementary file 1 — Figure S1. An architecture of Knomics-Biota web service. (PDF 31 kb) [file 13040_2018_187_MOESM1_ESM.pdf]
