# Supplementary material for: Knomics-Biota - a system for exploratory analysis of human gut microbiota data
Source: BioData Min. 2018 Nov 6;11:25. doi: 10.1186/s13040-018-0187-3 (PMC6220475; doi:10.1186/s13040-018-0187-3)

On each isolated figure, a single distinct pathway is shown in red.

Vitamin B7

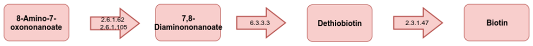

Vitamin B12

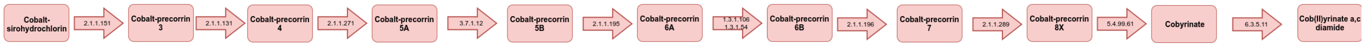

Vitamin B3

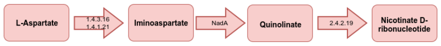

Vitamin K

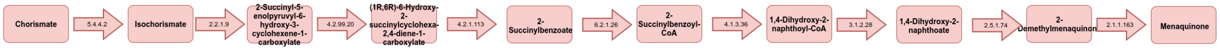

Vitamin B9

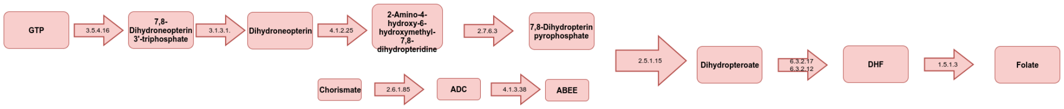

Vitamin B6 (pathway a)

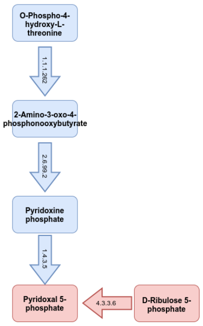

Vitamin B6 (pathway b)

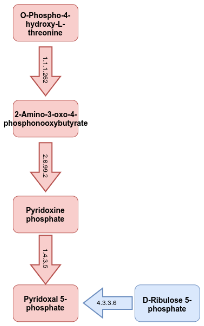

Vitamin B5

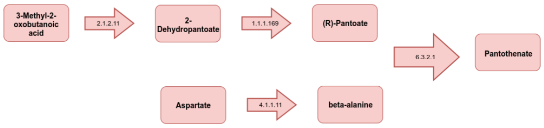

Vitamin B2

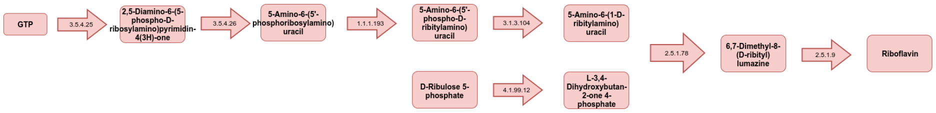

Vitamin B1 (pathway a)

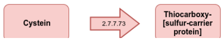

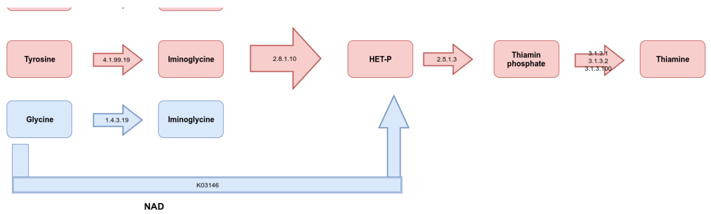

Vitamin B1 (pathway b)

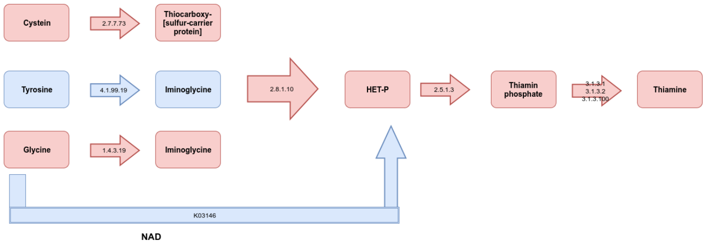

Vitamin B1 (pathway c)

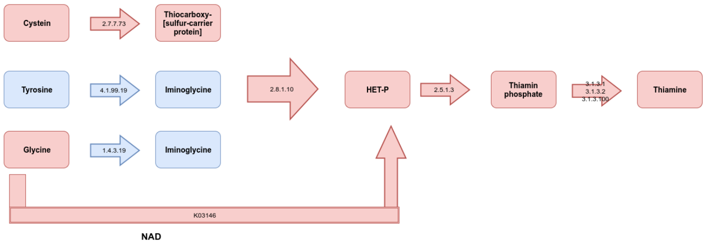

Butyrate (pathway Acetyl-CoA)

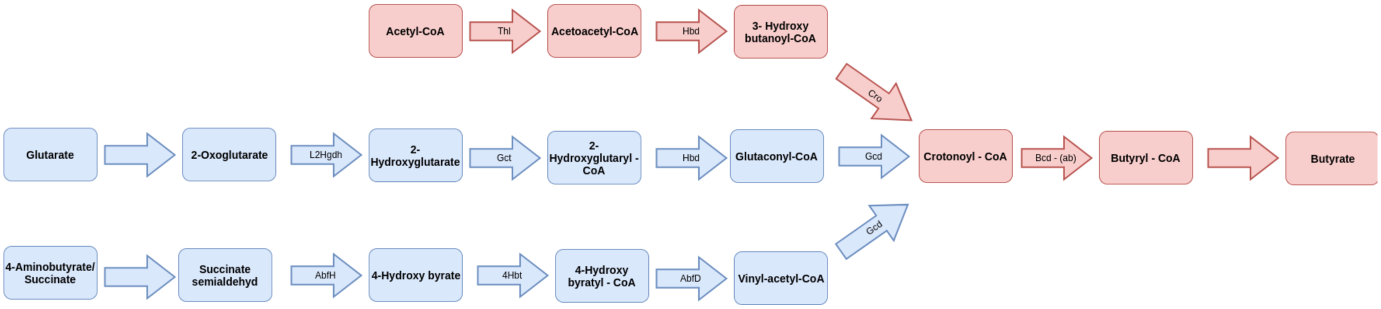

Butyrate (pathway Glutarate)

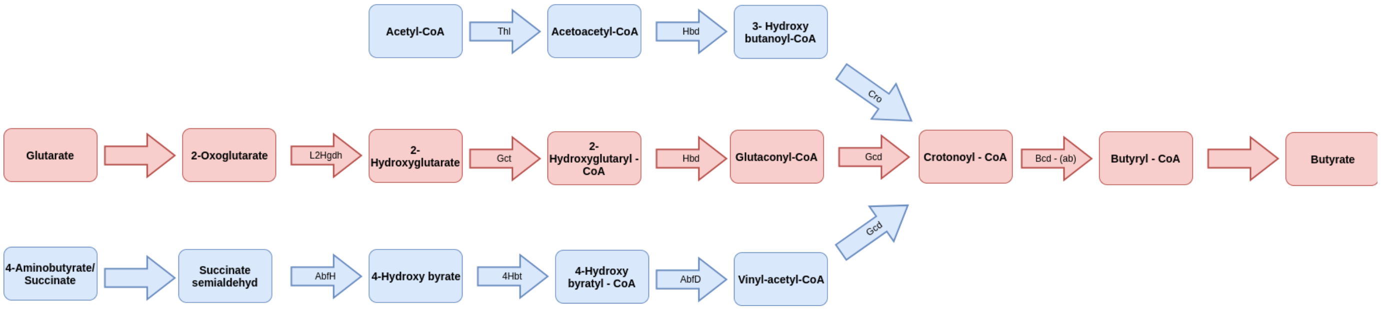

Butyrate (pathway 4-Aminobutyrate/Succinate)

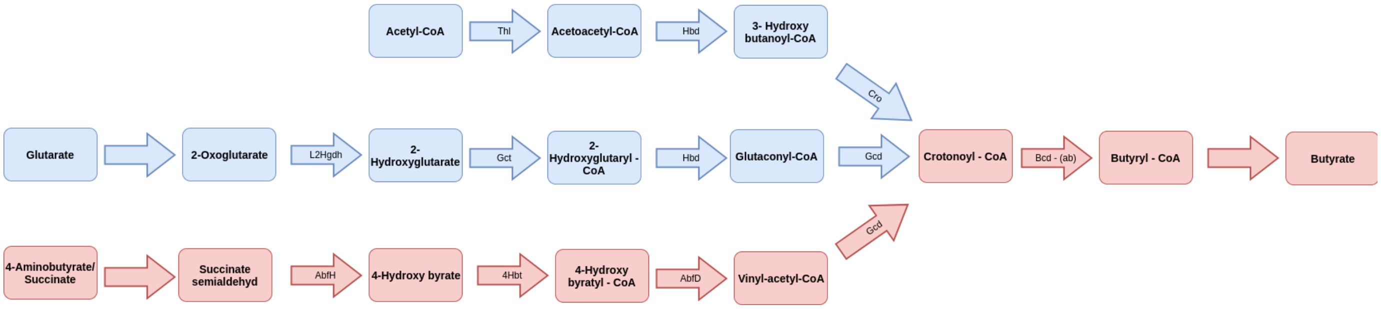

Propionate (pathway 1,2-Propanediol)

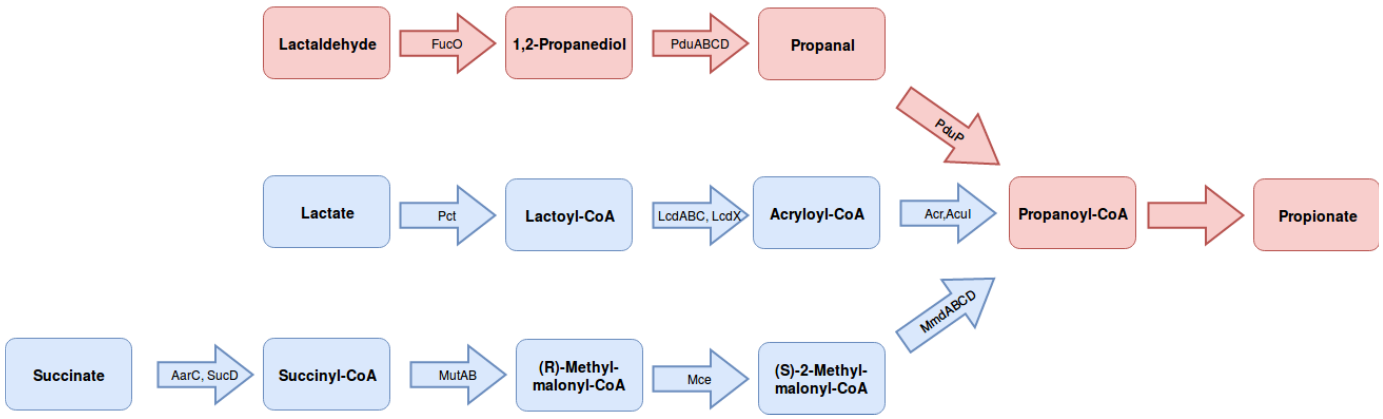

Propionate (pathway Acrylate)

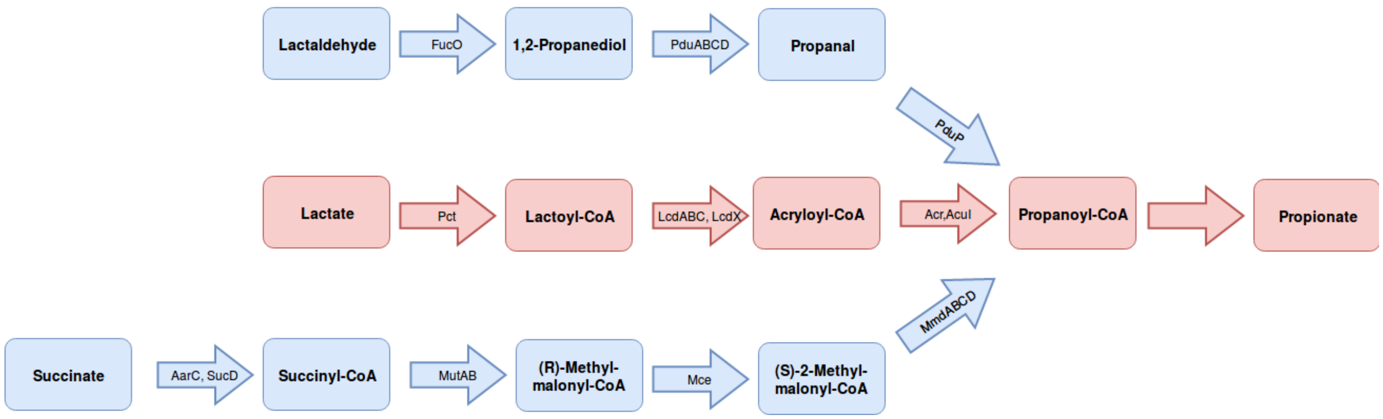

Propionate (pathway Succinate)

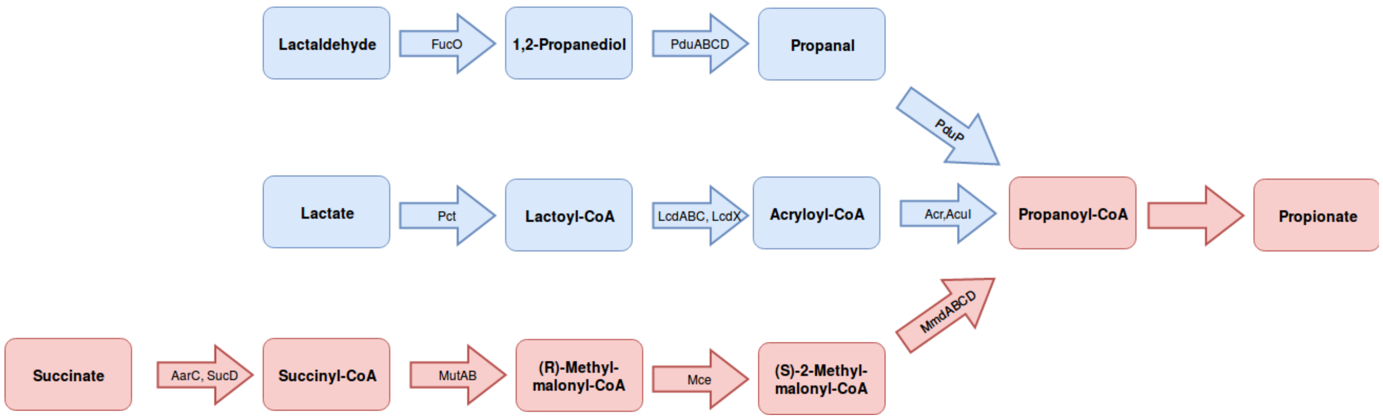

Supplement: Supplementary file 2 — Figure S2. Manually curated vitamin biosynthesis pathways used in the analysis. (PDF 1598 kb) [file 13040_2018_187_MOESM2_ESM.pdf]
